# Intravenous Tranexamic Acid for Control of Bleeding during External Dacryocystorhinostomy under General Anesthesia: A Randomized Clinical Trial

**DOI:** 10.18502/jovr.v19i3.13947

**Published:** 2024-09-16

**Authors:** Mohammad Sharifi, Mohammad Yaser Kiarudi, Samaneh Gholamhoseinpour-Omran, Mohammad Alipour, Elham Bakhtiari

**Affiliations:** ^1^Eye Research Center, Mashhad University of Medical Sciences, Mashhad, Iran; ^2^Department of Ophthalmology, Mashhad University of Medical Sciences, Mashhad, Iran; ^3^Eye Research Center, Department of Ophthalmology, Mashhad University of Medical Sciences, Mashhad, Iran; ^4^Clinical Research Unit, Mashhad University of Medical Sciences, Mashhad, Iran; ^6^https://orcid.org/0000-0003-1893-7849

**Keywords:** External Dacryocystorhinostomy, Intraoperative Hemorrhage, Intravenous Tranexamic Acid

## Abstract

**Purpose:**

To investigate the effect of intravenous tranexamic acid administered prior to external dacryocystorhinostomy (DCR) surgery to decrease intraoperative bleeding under general anesthesia.

**Methods:**

This was a double-blinded randomized placebo-controlled trial. A total of 70 patients (35 intervention and 35 control) with nasolacrimal duct obstruction (NLDO) who were selected for DCR surgery between September 2021 and September 2022 were included. After clinical examinations and laboratory tests, patients were randomly classified into intervention and control groups. The intervention group received 10 mg/kg intravenous tranexamic acid to a maximum dose of 1 gr 30 minutes before the surgery. Controls received normal saline solution as a placebo. The amount of intraoperative bleeding and surgical time were compared between the two groups.

**Results:**

The intervention group included 21 men (60%) and 14 women (40%), while the control group included 19 men (54.3%) and 16 women (45.7%). The mean ages of the participants were 55.46 ± 10.8 years and 58.06 ± 11.28 years in the intervention and control groups, respectively. A significant difference was observed between the two groups in the surgical time analysis (control group: 37.74 ± 9.52 minutes vs intervention: 26.03 ± 10.5 minutes; P < 0.001). Additionally, there was a significant difference in the bleeding volume between the intervention (70.66 ± 48.19 ml) and control (47.74 ± 60 ml) groups (P < 0.001).

**Conclusion:**

Intravenous tranexamic acid administration before the DCR procedure can successfully control bleeding during the surgery.

##  INTRODUCTION

Nasolacrimal duct obstruction (NLDO) is a common lacrimal drainage disorder that can be acquired or congenital. It typically presents with chronic epiphora, mucoid discharge, and sometimes acute or chronic dacryocystitis.^[1,2]^ Dacryocystorhinostomy (DCR) is the standard surgery for managing NLDO with a success rate of 54–95% and is performed with external or internal endonasal approaches.^[3,4]^

Intraoperative hemorrhage and damage to nasal mucosa are known complications of the external DCR.^[5,6]^ Hemorrhage can obstruct clear visualization during external DCR surgery.^[7]^ Blood pressure management, appropriate nasal packing, well-powered suction, and cautery are essential and additional tips for good hemostasis.^[8]^

Surgical factors such as general anesthesia and medications like aspirin, heparin, warfarin, and selective serotonin reuptake inhibitors (SSRI) might have a role in the stimulation of bleeding during the DCR surgery.^[4][5,6][9][10][11]^ It is well-known that some medications, such as tranexamic acid, an antifibrinolytic agent, would effectively reduce intraoperative hemorrhage during minor and major surgeries.^[12,13]^ Tranexamic acid is a synthetic drug that prevents plasminogen activation to plasmin competitively.^[14]^ To the best of the authors' knowledge, only a few studies have investigated the effect of medication in controlling bleeding during DCR surgery, thus the present study aims to better understand how intravenous tranexamic acid affects intraoperative hemorrhage in patients undergoing external DCR surgery.

##  METHODS 

This was a double-blinded, randomized placebo-controlled trial. The study was conducted on patients referred to the Khatam – Alanbia Eye Hospital, affiliated with Mashhad University of Medical Sciences, Mashhad, Iran, between September 2021 and September 2022. The study procedures have adhered to the Declaration of Helsinki, and the study details were approved by the Ethics Committee of Mashhad University of Medical Sciences (IR.MUMS.MEDICAL.REC.1400.520). Furthermore, study details have been registered on the website of https://fa.irct.ir/login, and its steps were registered via the registration number (IRCT 20211121053138 N 1). The randomized controlled trial adhered to the CONSORT flow diagram.

Seventy patients aged over 18 years with primary NLDO and no history of trauma were included in the study. Patients with a history of coronavirus contamination within the last six months or those with previous DCR, acute dacryocystitis, history of thrombosis, renal or liver diseases, systemic hypertension, cardiovascular diseases, anticoagulants consumers, and having allergic reactions to the drug were excluded.

Upon explaining the study procedures to eligible patients, all patients were initially asked to sign a written consent form. A follow-up interview was conducted to assess the history of ocular and systemic health conditions.

**Table 1:** Demographic characteristics of the study population.


**Factors**	**Level**	**Total**	**Groups**			**P-value**
			**Control**	**Intervention**	
Gender (%)	Female	30 (42.9%)	16 (45.7%)	14 (40.0%)	0.809*
	Male	40 (57.1%)	19 (54.3%)	21 (60.0%)	
					
Age (yrs)	Mean ± SD	56.76 ± 11.04	58.06 ± 11.28	55.46 ± 10.8	0.328**
	Median (Range)	57.5 (36 to 78)	59 (36 to 78)	56 (38 to 75)	
	
	
SD, standard deviation; yrs, years ${\;}^{*}$ *P*-value based on Fisher Exact test; ${\;}^{**}$ *P*-value based on *T*-test									

**Table 2:** The mean value of the blood factors in the case and control groups.


**Factors**	**Total**	**Groups**			***P*** **-value**
		**Control**	**Intervention**	
PT	12.34 ± 0.34	12.33 ± 0.33	12.35 ± 0.36	0.961 ${\;}^{*}$
	12.2 (12 to 13)	12.2 (12 to 13)	12.2 (12 to 13)	
				
INR	1.08 ± 0.08	1.08 ± 0.08	1.08 ± 0.08	0.666 ${\;}^{*}$
	1.06 (1 to 1.24)	1.06 (1 to 1.24)	1.06 (1 to 1.22)	
				
Plt	266157.14 ± 74682.06	253114.29 ± 75375.83	279200 ± 72712.49	0.111 ${\;}^{**}$
	262000 (127000 to 439000)	228000 (127000 to 421000)	272000 (157000 to 439000)	
	
	
INR, international normalized ratio; Plt, platelets; PT, prothrombin time ${\;}^{*}$ *P*-value based on Mann-Whitney test; ${\;}^{**}$ *P*-value based on *T*-test							

**Table 3:** The mean value of intraoperative hemorrhage volume and duration of surgery in the case and control groups.


**Factors**	**Total**	**Groups**			***P*** **-value**
		**Control**	**Intervention**	
Bleeding volume (cc)	59.2 ± 55.24	70.66 ± 48.19	47.74 ± 60	< 0.001
	42 (11 to 274)	56 (25 to 274)	29 (11 to 274)	
				
Duration of surgery (min)	36.89 ± 9.99	37.74 ± 9.52	26.03 ± 10.5	< 0.001
	34 (24 to 70)	36 (27 to 70)	34 (24 to 70)	
	
	
min, minutes ${\;}^{*}$ *P*-value based on Mann-Whitney test							

### Clinical and Laboratory Testing

Patients were initially examined using a slit lamp to check the ocular discharge, epiphora, and lacrimal drainage system. Then, they were examined using diagnostic lacrimal irrigation tests to detect the NLDO. If the patient had reflux of fluid from another punctum during irrigation of canaliculi with syringe and lacrimal cannula, NLDO was considered.

If patient had canalicular obstruction proved by inability to pass lacrimal probe via canaliculi or soft touch encountered during diagnostic probing, then common canaliculi obstruction was considered. Both conditions were excluded in the study

DCR had incision on skin, orbicularis muscle, and nasal mucosa with rich blood supply. Some patients have undiagnosed bleeding tendency disorder that might cause profound bleeding during DCR surgery. So, a protocol is used to investigate and check PTT, PT, and platelet function and count before DCR in our hospital. In the present study, prior to the DCR surgery, all patients underwent laboratory testing to check the blood coagulation profile by using the analysis of the blood factors of prothrombin time (PT), platelets (Plt), and international normalized ratio (INR). Then, the patients were randomly classified into the intervention and control groups. The intervention group received 10 mg/kg of intravenous tranexamic acid up to a maximum dose of 1 gr 30 min before the DCR surgery. Controls were injected with normal saline solution as a placebo.

### Randomizations

The permuted-block randomization method was applied with a random block length between two and six. The random allocation sequence was generated using a computer program and concealed from the investigators. Both surgeon and anesthetist who measured bleeding volume were masked.

### Main Outcome Measures

Intraoperative hemorrhage during the DCR surgery was considered the primary outcome. A calibrated suction device measured intraoperative hemorrhage after deducting the normal saline solution. The time of surgery was defined as the secondary outcome measure.

### General Anesthesia and Dacryocystorhinostomy (DCR) Protocol

Local anesthesia had less bleeding than general anesthesia which was shown in literature.

Some ophthalmologists use general anesthesia for DCR, and we wanted to use a medication that can show the bleeding amount and the operation time, and then compare it with the standard protocol. Bleeding volume and operation time are the two main concerns in DCR. Medication to control bleeding during DCR may reduce operation time and increase surgeon comfort during surgery.

Anesthesia was administered to all patients in the same way. The patients were placed on an operating room bed and given an IV line. Standard monitoring was established for the patient, and Ringer's serum was prescribed at 5 cc/kg. The intervention group received 10 mg/kg of intravenous tranexamic acid up to a maximum dose of 1 gr 30 minutes before the DCR surgery. In contrast, the control group received a normal saline solution as a placebo. Anesthesia induction, which included midazolam 0.015 mg/kg, fentanyl 2 μ g/kg, propofol 2–3 mg/kg, and atracurium 0.5 mg/kg, was applied. Patients also received 100–150 μ g/kg propofol and 0.6–0.8 isoflurane for maintenance. All patients received oral 0.5 mg alprazolam for premedication the night before surgery and 2 hr before the DCR surgery. The anesthetist had control of blood pressure and heart rate during the surgery (systolic no more than 120 mmHg, diastolic no more than 80 mmHg, heart rate no more than 100 per minute). We used some head elevation for better visualization and surgeon convenience in all patients. We did not use cautery, local lidocaine, and epinephrine.

After a curvilinear skin incision 3–4 mm from the medial canthus and 10–12 mm in length along the anterior lacrimal crest, blunt dissection of the orbicularis muscle was performed to reach the periosteum. Then lacrimal fossa exposure, bone punching, lacrimal sac, and nasal mucosal flap creation were done, respectively. Two flaps were opposed edge to edge, followed by orbicularis and skin suturing. An experienced oculoplastic surgeon (MS) did all the surgeries. Intraoperative blood loss was calculated by deducting the saline solution used to clean the surgical site from the total bloody liquid aspirated in the scaled suction machine at the end of each surgery.^[10]^ The bottle attached to the suction machine was scaled at 5 cc intervals. The bottle had a capacity of 50 cc, and if the bleeding volume was more than 50 cc, a second bottle was used. The amount of solution for irrigation was subtracted from the bottle volume at the end of surgery. To prevent aspiration and show nasopharynx bleeding, gauze was put in the throat. We did not use any swabs to mop the bleeding. The amount of blood loss into the nasopharynx during extubation was too little so we did not show it in the calculation.

### Sample Size

Following Beikaei et al's study, the sample size was calculated according to the volume of blood loss in patients undergoing elective rhinoplasty surgery with and without tranexamic acid injection.^[7]^ The volume of blood loss in patients without tranexamic acid injection was 60.3 ± 9.5 ml and in patients with tranexamic acid injection was 43.3 ± 11 ml, respectively. Considering a drop-out rate of 15%, the sample size of 35 patients in each group was calculated ( α = 0.05 and power = 0.95).

### Statistical Analysis

Data were analyzed using the SPSS 16 (SPSS Inc., Chicago, Illinois, United States). Normal distribution was examined using the Kolmogorov–Smirnov test. Fisher's exact test or Chi-square was used to determine the association of categorical variables. Quantitative variables were compared using an independent-sample *T*-test or non-parametric equivalent. A *P*-value < 0.05 was considered statistically significant.

##  RESULTS

A total of 70 individuals participated in the present study, including 35 participants in the intervention and 35 participants in the control group. The mean age ± standard deviation of all participants was 56.76 ± 11.04 years, and 57.1% were male. There were no significant differences in terms of age and gender between the two groups [Table 1].

The blood coagulation profile in both the intervention and control groups is shown in Table 2. There was no significant difference between the two groups regarding the blood factors of prothrombin time (control: 12.33 ± 0.33 vs intervention: 12.35 ± 0.36), international normalized ratio (control: 1.08 ± 0.08 vs intervention: 1.08 ± 0.08), and platelet count (control: 253114.29 ± 75375.83 vs intervention: 279200 ± 72712.49).

As shown in Table 3, there was a significant difference between the two groups in the analysis of the time of surgery (control: 37.74 ± 9.52 vs intervention: 26.03 ± 10.5 minutes). A significant difference was observed in terms of bleeding volume – control group: 70.66 ± 48.19 ml in comparison with NLDO patients who received intravenous tranexamic acid in the case group: 47.74 ± 60 ml (*P* < 0.001).

##  DISCUSSION 

During external DCR surgery, the primary surgical concern that impairs the surgeons' vision is intraoperative hemorrhage.^[6,10]^

Tranexamic acid (Trans -4-(Amino methyl) cyclohexane carboxylic acid) is a synthetic drug driven from the amino acid lysine that prevents plasminogen activation to plasmin competitively.^[14]^ This medication might be activated directly by binding to Kringle chain regions. The time taken for drug concentration in the human body has been estimated at 120 minutes, which could transfer to all body organs.^[15,16]^ Today, it has been widely applied for intraoperative usages to reduce bleeding and mortality rates during cardiovascular or other surgeries.^[16]^ Patients' gender, weight, and surgery duration can affect the hemostatic property of tranexamic acid.^[8]^

It has been found that this medication could reduce transfusion and is also applicable for topical usage.^[17]^ It has been reported that this type of medication might be associated with limited complications except in high dosages.^[15,16][17]^ This drug should be used cautiously in neurologic patients due to the possible interaction with the Gamma-Aminobutyric Acid (GABA) and glycine receptors.^[15,16][17]^ Clinical studies have shown fewer thrombotic complications and less risk of myocardial infarction in patients undergoing tranexamic acid therapy.^[15,16][17]^ Generally, seizure, orthostatic hypotension, diarrhea, and nausea have been reported as the most familiar side effects that might occur in cases of high dosages.^[18]^

Evidence showed the efficacy of tranexamic acid in surgeries such as endoscopic sinus surgery, arthroscopic shoulder surgery, and elective degenerative spine surgery.^[19,20,21]^ In rhinoplasty, it is safe and effective in decreasing intraoperative bleeding, postoperative eyelid edema, and ecchymosis.^[22]^

There are a few studies investigating the medication's effect in controlling bleeding during DCR surgery.^[12,13]^ In a randomized clinical trial conducted by Alam et al^[12]^ on 96 patients with NLDO who underwent DCR surgery, similar to our study, intraoperative blood loss was calculated by measuring the amount of blood aspirated in the suction machine during the surgery. The premeasured amount of saline used during surgery was subtracted from the total amount of blood collected in the suction bottle to roughly get the exact amount of blood loss. No significant difference was observed between the two groups regarding the time of surgery, which was contradictory to our findings. However, the time of surgery was shorter in the present study (control: 37.74 ± 9.52 minutes vs intervention: 26.03 ± 10.5 minutes) compared to the study by Alam et al^[12]^ (control: 53.38 ± 19.8 vs intervention: 48.43 ± 20.01 minutes). In addition, a case series study was conducted by Moradi Farsani et al^[13]^ on 30 candidate patients for DCR, whose bleeding volume was measured by deducting the saline volume used for irrigating from the total volume of fluid collected in the suction bottle 2 hr after the surgery. Gender, age, and the study drug group were considered independent parameters. The duration of surgery was 72.4 ± 15.7 minutes. This difference might have resulted from the surgeon's experience; in our study, surgeries were done by an experienced oculoplastic surgeon.

We found that the bleeding volume was considerably lower in the intervention groups that received preoperative intravenous tranexamic acid (control: 70.66 ± 48.19 ml vs intervention: 47.74 ± 60 ml). However, no difference was reported in the study by Alam et al^[12]^, resulting in 88.63 ± 69.34 ml in the intervention group versus 88.89 ± 51.93 ml in the control group. Overall, the bleeding volume was higher in the study by Alam et al^[12]^ in both groups, which could have resulted from the longer duration of surgery. Furthermore, the bleeding volume was remarkably greater (146.83 ± 91 ml) in the study by Moradi Farsani et al^[13]^, which might be related to the longer time of DCR surgery and drug injection protocol (the patients received tranexamic acid over 15 minutes before the surgery intravenous infusion, that might not reach to peak serum level at the beginning of the surgery). These studies evaluated intraoperative and early postoperative hemorrhage, however, in our study, we only measured intraoperative bleeding which might be the reason for a different result. Although we did not include nasal packing in our study because our focus was intraoperative bleeding, we found a significant difference in the duration of surgery between the intervention and control groups that could be attributed to less blood loss in the intervention group. We did not observe any complications related to tranexamic acid injection such as seizure, embolic phenomena, and anaphylaxis in our study. There were no significant differences between the three studies concerning population demographic characteristics, surgical technique, and protocol of general anesthesia.

Systemic hypertension, blood, liver, renal disorders, and anesthesia agents may be considered confounders of this study but we used the same protocol of anesthesia for individuals and excluded patients with liver, renal, and blood disorders from the study. The hypotensive and hypertensive state was monitored during surgery by the anesthetist so all patients had hemodynamic stability during the surgery.

The strengths of the present study were the study design and the examination of blood factors. The latter alleviates surgeon's concern at the time of intraoperative massive bleeding.^[23]^ Whereas, the study's limitation can be attributed to the limited number of sample size.

In conclusion, preoperative use of intravenous tranexamic acid could successfully control the bleeding volume during external DCR surgery in patients with primary NLDO.

### Financial Support and Sponsorship

None.

### Conflicts of Interest

None.
